# The impact of fasting stress hyperglycemia ratio, fasting plasma glucose and hemoglobin A1c on in-hospital mortality in patients with and without diabetes: findings from the China acute myocardial infarction registry

**DOI:** 10.1186/s12933-023-01868-7

**Published:** 2023-07-04

**Authors:** Kongyong Cui, Rui Fu, Jingang Yang, Haiyan Xu, Dong Yin, Weihua Song, Hongjian Wang, Chenggang Zhu, Lei Feng, Zhifang Wang, Qingsheng Wang, Ye Lu, Kefei Dou, Yuejin Yang, the CAMI Registry Investigators

**Affiliations:** 1 Cardiometabolic Medicine Center, Department of Cardiology, National Center for Cardiovascular Diseases, State Key Laboratory of Cardiovascular Disease, National Clinical Research Center for Cardiovascular Diseases, FuWai Hospital, Chinese Academy of Medical Sciences and Peking Union Medical College, No. 167, Beilishi Road, Xicheng District, Beijing, 100037 China; 2 Coronary Heart Disease Center, Department of Cardiology, National Center for Cardiovascular Diseases, State Key Laboratory of Cardiovascular Disease, National Clinical Research Center for Cardiovascular Diseases, FuWai Hospital, Chinese Academy of Medical Sciences and Peking Union Medical College, No. 167, Beilishi Road, Xicheng District, Beijing, 100037 China; 3 Department of Cardiology, Xinxiang Central Hospital, The Fourth Clinical College of Xinxiang Medical University, Xinxiang, Henan Province China; 4 Department of Cardiology, Qinhuangdao First Hospital, Qinhuangdao, Hebei Province China; 5 Medical Research & Biometrics Center, FuWai Hospital, National Center for Cardiovascular Diseases, Chinese Academy of Medical Sciences and Peking Union Medical College, Beijing, China

**Keywords:** Fasting stress hyperglycemia ratio, Fasting plasma glucose, Hemoglobin A1c, Acute myocardial infarction, Glucose metabolism status, In-hospital mortality

## Abstract

**Background:**

Stress hyperglycemia was positively associated with poor prognosis in individuals with acute myocardial infarction (AMI). However, admission glucose and stress hyperglycemia ratio (SHR) may not be the best indicator of stress hyperglycemia. We performed this study to evaluate the comparative prognostic value of different measures of hyperglycemia (fasting SHR, fasting plasma glucose [FPG], and hemoglobin A1c [HbA1c]) for in-hospital mortality in AMI patients with or without diabetes.

**Methods:**

In this prospective, nationwide, multicenter China Acute Myocardial Infarction (CAMI) registry, 5,308 AMI patients including 2081 with diabetes and 3227 without diabetes were evaluated. Fasting SHR was calculated using the formula [(first FPG (mmol/l))/(1.59×HbA1c (%)-2.59)]. According to the quartiles of fasting SHR, FPG and HbA1c, diabetic and non-diabetic patients were divided into four groups, respectively. The primary endpoint was in-hospital mortality.

**Results:**

Overall, 225 (4.2%) patients died during hospitalization. Individuals in quartile 4 had a significantly higher rate of in-hospital mortality compared with those in quartile 1 in diabetic cohort (9.7% vs. 2.0%; adjusted odds ratio [OR] 4.070, 95% CI 2.014–8.228) and nondiabetic cohort (8.8% vs. 2.2%; adjusted OR 2.976, 95% CI 1.695–5.224). Fasting SHR was also correlated with higher in-hospital mortality when treated as a continuous variable in diabetic and nondiabetic patients. Similar results were observed for FPG either as a continuous variable or a categorical variable. In addition, fasting SHR and FPG, rather than HbA1c, had a moderate predictive value for in-hospital mortality in patients with diabetes (areas under the curve [AUC] for fasting SHR: 0.702; FPG: 0.689) and without diabetes (AUC for fasting SHR: 0.690; FPG: 0.693). The AUC for fasting SHR was not significantly different from that of FPG in diabetic and nondiabetic patients. Moreover, adding fasting SHR or FPG to the original model led to a significant improvement in C-statistic regardless of diabetic status.

**Conclusions:**

This study indicated that, in individuals with AMI, fasting SHR as well as FPG was strongly associated with in-hospital mortality regardless of glucose metabolism status. Fasting SHR and FPG might be considered as a useful marker for risk stratification in this population.

*Trial registration*: ClinicalTrials.gov NCT01874691.

**Supplementary Information:**

The online version contains supplementary material available at 10.1186/s12933-023-01868-7.

## Introduction

In clinical practice, stress hyperglycemia is positively associated with poor prognosis in individuals with critical illnesses such as acute myocardial infarction (AMI) [1], heart failure [2], and stroke [3]. Currently, there is no consensus on the definition of stress hyperglycemia in patients with AMI. Most early studies defined hyperglycemia by the first acquired glucose value at admission. However, previous studies reported that admission glucose values were positively associated with short- and long-term mortality in individuals without diabetes [4–10], whereas in those with established diabetes, this was not the case [5, 8–10]. Stress hyperglycemia ratio (SHR) was defined as admission glucose divided by the estimated average glucose, which was derived from glycosylated hemoglobin A1c (HbA1c) [11]. Theoretically, it could identify stress hyperglycemia more accurately by adjusting the chronic glycemic status of the past 2 ~ 3 months. Some studies, including ours, showed a significant association between SHR and mortality in patients with AMI [12–14]. Marenzi and colleagues found that SHR was a better predictor of in-hospital morbidity and mortality than admission glucose in AMI patients with diabetes [12]. Chen and colleagues reported that SHR was an independent predictor of in-hospital mortality for patients with AMI even after adjusting for the Global Registry of Acute Coronary Events (GRACE) score [13]. Our previous study revealed that SHR was positively associated with 2-year mortality in AMI patients with or without diabetes [14]. Nonetheless, the Singapore Myocardial Infarction Registry reported that no significant association was found between SHR and 1-year mortality in patients with non-ST-segment elevation myocardial infarction (NSTEMI) [15]. Moreover, a study with 6287 patients with ST-segment elevation myocardial infarction (STEMI) revealed that the highest SHR quartile was not significantly associated with higher mortality in diabetic patients [16].

Actually, in addition to acute stress condition and chronic glycemic levels, admission glucose values are also subject to meal timing. Aronson and colleagues found that elevated fasting plasma glucose (FPG) was superior to admission glucose in predicting 30-day mortality in 735 nondiabetic patients with AMI [17]. In addition, we have previously shown a strong positive association between fasting SHR (calculated with first FPG and HbA1c) and in-hospital mortality in patients with AMI irrespective of glucose metabolism status [18]. In this study, we used the data from China Acute Myocardial Infarction (CAMI) registry to evaluate the comparative prognostic value of different measures of hyperglycemia (fasting SHR, FPG, and HbA1c) for in-hospital mortality in AMI patients with different glucose metabolism status.

## Materials and methods

### Study design and population

This was an analysis of the prospective, nationwide, multicenter CAMI registry, and the study design has been described in existing literatures [14, 19, 20]. The study was registered on www.Clinicaltrials.gov (NCT01874691). Overall, 108 hospitals including 31 provincial hospitals, 45 municipal hospitals, and 32 county hospitals throughout China had participated in the registry since January 2013. In Phase I, patients with type 1, 2, 3, 4b, or 4c of STEMI or NSTEMI who were admitted ≤ 7 days of symptom onset were consecutively enrolled. However, only individuals admitted ≤ 3 days of symptom onset were registered from the participating hospitals from September, 2014 to January, 2016 (Phase II). During hospitalization, the participants received optimal medical therapy and/or coronary revascularization according to the recommendations of contemporary guidelines, cardiologist’s discretion and their own preference. The members of committees and a complete list of investigators are listed in Additional file 1: Tables S1 and S2, respectively. The registry was performed in compliance with the principles of the Declaration of Helsinki and was approved by the Institutional Review Board of each participating hospital. All the participants provided written informed consent before enrollment.

**Fig. 1:**
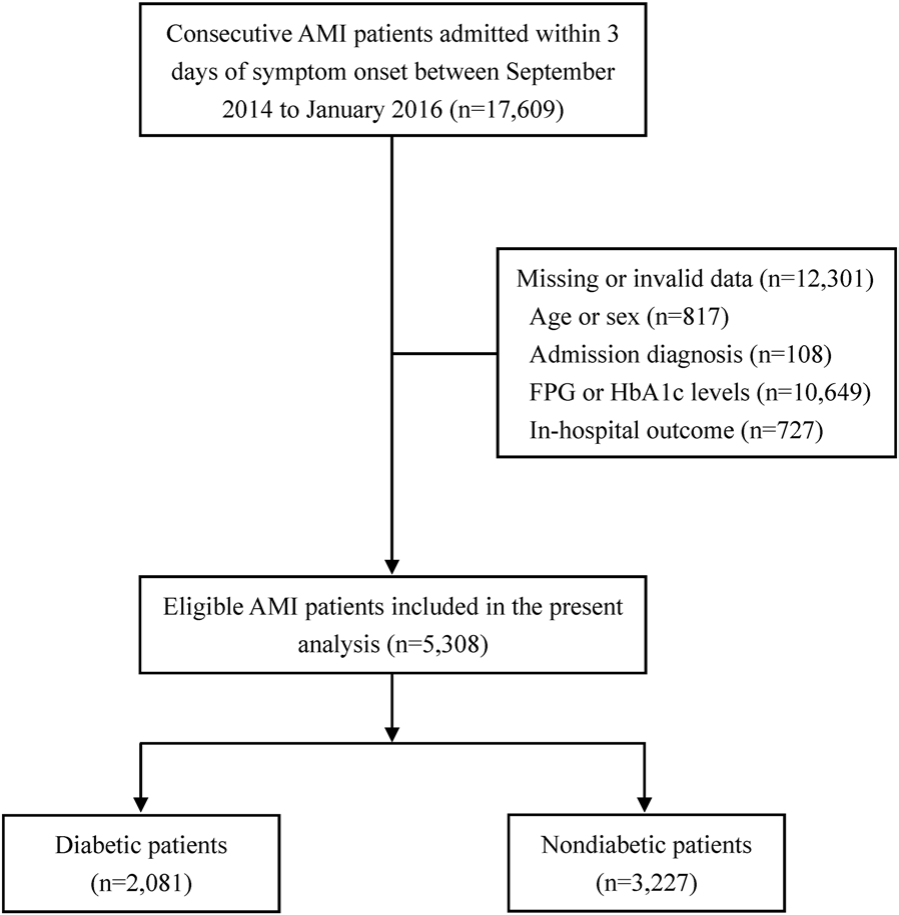
Flow chart of the study. *AMI* acute myocardial infarction, *FPG* fasting plasma glucose, *HbA1c* hemoglobin A1c

The present analysis was based on Phase II of the CAMI registry, which registered a total of 17,609 patients with AMI. Of note, patients with missing or invalid data on age or sex (n = 817), diagnosis (n = 108), concentrations of FPG or HbA1c (n = 10,649), and in-hospital outcomes (n = 727) were excluded. As a result, we analyzed 5,308 patients with AMI who met the selection criteria **(**Fig. 1**)**.

### Data collection and definitions

Demographics, cardiovascular risk factors, clinical parameters, laboratory results, imaging findings, reperfusion details, and medications were prospectively collected with standardized questionnaires. Data were collected, validated and submitted through a secure, password-protected, web-based electronic data capture system by well-trained independent investigators in each participating hospital. Once a patient admitted to emergency department met the inclusion criteria, the front page of electronic case report form (eCRF) would be filled out and submitted online within 24 h from admission. The investigators should collect all the data during hospitalization and submit the completed eCRF upon the patient’s discharge or death.

Diabetes was defined as having a history of diabetes, receiving hypoglycemic therapy before admission, or having HbA1c levels ≥ 6.5% at admission [21]. Hypertension was defined as systolic blood pressure ≥ 140 mmHg, diastolic blood pressure ≥ 90 mmHg, or use of antihypertensive treatment before admission [22]. Hyperlipidemia was defined as plasma triglyceride ≥ 200 mg/dl, total cholesterol ≥ 240 mg/dl, or use of lipid-lowering drugs before admission [23].

### Endpoints

The primary endpoint was in-hospital death, including cardiac or non-cardiac death during hospitalization. All deaths were adjudicated by medical personnel who were not investigators in this study and who were blinded to the clinical and laboratory data of the patients, based on death certificates, hospital record abstracts and related reports (autopsy, biopsy, and diagnostic output).

### Statistical analysis

Fasting SHR was calculated by the formula [(first FPG (mmol/l))/(1.59×HbA1c (%) -2.59)] [11, 18]. According to the quartiles of fasting SHR, FPG and HbA1c, diabetic and non-diabetic patients were divided into four groups, respectively. Continuous variables were expressed as mean ± standard deviation if they were the normal distribution, otherwise as median (interquartile range). We used ANOVA to plot the differences of continuous variables among different groups. The Kruskal-Wallis H test was applied for non-normally distributed continuous variables. Categorical variables were expressed as frequencies (percentages) and compared using Pearson’s Chi-square test or Fisher’s exact test, when appropriate. To evaluate the prognostic effect of fasting SHR, single-variable and multivariable logistic regression analysis were performed, and odds ratios (ORs) with 95% confidence intervals (CIs) were calculated. In multivariable analysis, we included clinically important factors and statistically significant variables in the single-variable analysis.

In addition, the receiver operating characteristic (ROC) curve analysis were performed and areas under the curve (AUC) were calculated to evaluate the predictive value of fasting SHR, FPG and HbA1c for in-hospital mortality [24]. The values were interpreted with the following standard: negligible (≤ 0.55), small (0.56–0.63), moderate (0.64–0.70), and strong (≥ 0.71). Harrell’s C-statistic was calculated to evaluate whether introducing fasting SHR, FPG or HbA1c into in a model of traditional risk factors could improve the predictive value [25]. All statistical analyses were performed in SPSS version 23.0 (SPSS Inc., Chicago, IL, USA) and R version 3.6.0 (R Foundation for Statistical Computing, Vienna, Austria). A two-sided P value of < 0.05 was considered statistically significant.

## Results

### Baseline characteristics

Of the 2081 diabetic patients, the mean age was 63.1 years, the mean BMI was 25.0 kg/m^2^, 68.2% were male, 38.1% were current smokers, 62.7% had hypertension, 16.1% had hyperlipidemia, 72.7% presented with STEMI, 24.4% had Killip class II/III/IV, and 35.8% underwent primary percutaneous coronary intervention (PCI) **(**Table 1**)**. We found statistically significant differences among the four groups in age, sex, hyperlipidemia, chronic kidney disease, clinical diagnosis, anterior myocardial infarction (MI), Killip class II/III/IV, heart rate, left ventricular ejection fraction (LVEF), levels of triglyceride, FPG and HbA1c, and use of angiotensin converting enzyme inhibitor/angiotensin receptor blocker during hospitalization. From quartile 1 to quartile 4, there was an ascending gradient with respect to anterior MI, Killip class II/III/IV, heart rate and FPG levels, whereas there was a descending gradient regarding LVEF (Additional file 1: Table S3).

**Table 1:** Baseline characteristics of the study population

Variable	Overall population (n = 5308)	Diabetes (n = 2081)	No diabetes (n = 3227)
Age (years)	62.0 ± 12.4	63.1 ± 11.5	61.3 ± 12.9
Male, n (%)	3961 (74.6)	1420 (68.2)	2541 (78.7)
Body mass index (kg/m^2^)	24.8 ± 3.0	25.0 ± 3.2	24.7 ± 2.9
Current smoking, n (%)	2474 (46.6)	792 (38.1)	1682 (52.1)
Hypertension, n (%)	2924 (55.1)	1304 (62.7)	1620 (50.2)
Hyperlipidemia, n (%)	778 (14.7)	336 (16.1)	442 (13.7)
Previous myocardial infarction, n (%)	370 (7.0)	194 (9.3)	176 (5.5)
Family history of premature CAD, n (%)	184 (3.5)	74 (3.6)	110 (3.4)
Previous PCI, n (%)	208 (3.9)	126 (6.1)	82 (2.5)
Previous CABG, n (%)	21 (0.4)	14 (0.7)	7 (0.2)
Previous stroke, n (%)	510 (9.6)	231 (11.1)	279 (8.6)
Peripheral vascular disease, n (%)	68 (1.3)	29 (1.4)	39 (1.2)
Previous heart failure, n (%)	127 (2.4)	71 (3.4)	56 (1.7)
Chronic kidney disease in treatment, n (%)	81 (1.5)	43 (2.1)	38 (1.2)
COPD, n (%)	104 (2.0)	41 (2.0)	63 (2.0)
STEMI, n (%)	4090 (77.1)	1512 (72.7)	2578 (79.9)
Anterior myocardial infarction, n (%)	2806 (52.9)	1061 (51.0)	1745 (54.1)
Killip class II/III/IV, n (%)	1183 (22.3)	508 (24.4)	675 (20.9)
Primary PCI, n (%)	2072 (39.0)	745 (35.8)	1327 (41.1)
Heart rate (beats/min)	79 ± 18	81 ± 18	78 ± 18
Systolic blood pressure (mmHg)	130 ± 24	132 ± 25	128 ± 24
Left ventricular ejection fraction (%)	53.6 ± 10.0	53.1 ± 10.2	53.9 ± 9.9
Laboratory data			
Triglyceride (mmol/L)	1.39 (0.99–2.02)	1.55 (1.11–2.33)	1.30 (0.93–1.82)
LDL-C (mmol/L)	2.71 (2.17–3.32)	2.71 (2.17–3.37)	2.71 (2.17–3.30)
HDL-C (mmol/L)	1.04 (0.89–1.22)	1.03 (0.86–1.18)	1.04 (0.90–1.24)
Fasting blood glucose (mmol/L)	6.86 (5.60–9.23)	9.32 (7.20-12.99)	6.06 (5.25–7.24)
HbA1c (%)	5.9 (5.5–7.1)	7.6 (6.7-9.0)	5.6 (5.3–5.9)
Serum creatinine (µmol/L)	75.1 (64.2–88.4)	75.1 (63.5–90.1)	75.1 (65.0-87.3)
Hemoglobin (g/L)	137 (124–149)	135 (122–148)	137 (125–149)
Medications during hospitalization			
Aspirin, n (%)	5081 (95.7)	2008 (96.5)	3073 (95.2)
P2Y_12_ inhibitor, n (%)	5214 (98.2)	2050 (98.5)	3164 (98.0)
ACEI/ARB, n (%)	3296 (62.1)	1295 (62.2)	2001 (62.0)
β-blockers, n (%)	4082 (76.9)	1621 (77.9)	2461 (76.3)
Statins, n (%)	5123 (96.5)	2015 (96.8)	3108 (96.3)

*ACEI* angiotensin converting enzyme inhibitor, *ARB* angiotensin receptor blocker, *CABG* coronary artery bypass grafting, *CAD* coronary artery disease, *COPD* chronic obstructive pulmonary disease, *HbA1c* glycosylated hemoglobin A1c, *HDL-C* high density lipoprotein cholesterol, *LDL-C* low-density lipoprotein cholesterol, *PCI* percutaneous coronary intervention, *STEMI* ST-segment elevation myocardial infarction

Among the 3227 nondiabetic patients, the mean age was 61.3 years, the mean BMI was 24.7 kg/m^2^, 78.7% were male, 52.1% were current smokers, 50.2% had hypertension, 13.7% had hyperlipidemia, 79.9% presented with STEMI, 20.9% had Killip class II/III/IV, and 41.1% underwent primary PCI **(**Table 1**)**. From quartile 1 to quartile 4, there was an ascending gradient in terms of FPG levels, whereas there was a descending gradient regarding the percentage of male and current smokers, LVEF, and HbA1c levels. In addition, age, percentages of individuals with previous stroke and Killip class II/III/IV, and heart rate were the highest in quartile 4 (Additional file 1: Table S4).

### Association between fasting SHR, FPG, HbA1c and in-hospital mortality

**Fig. 2:**
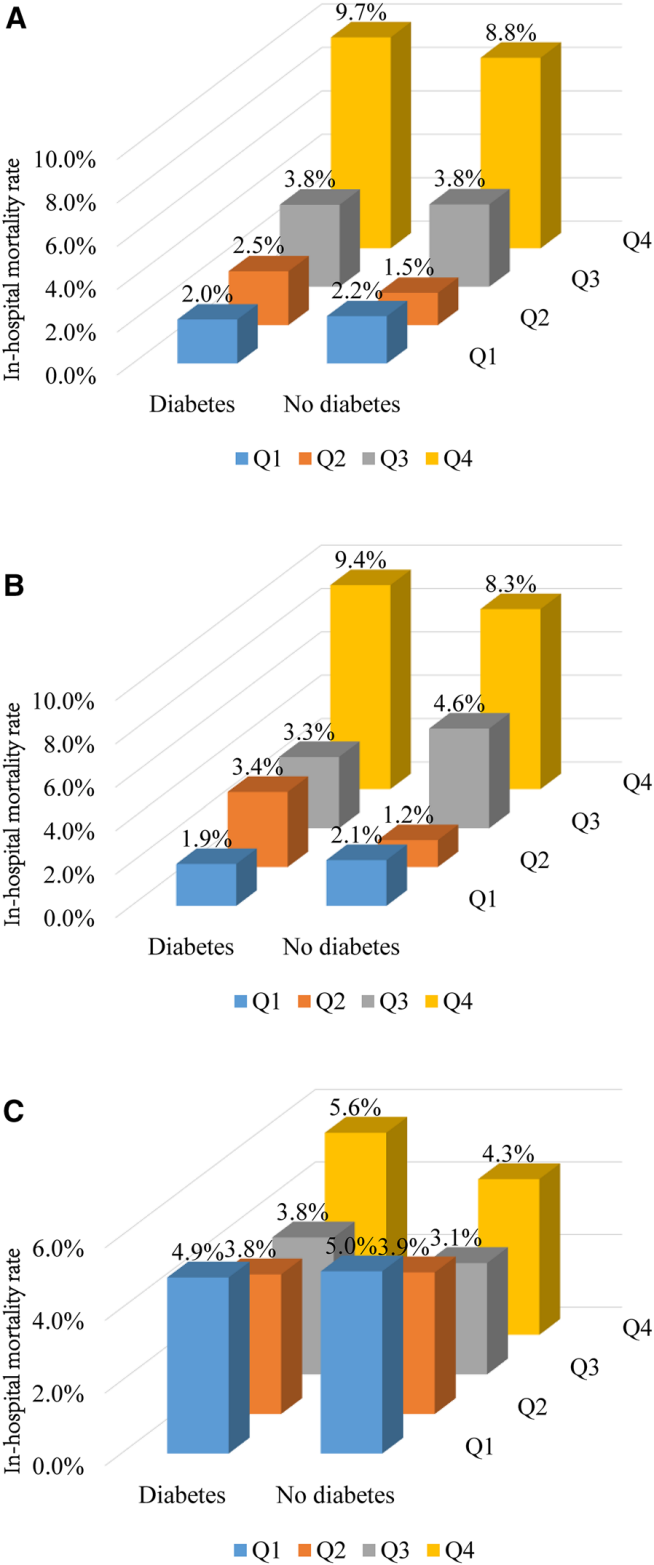
Rates of in-hospital mortality according to diabetes status and quartiles of **A** fasting stress hyperglycemia ratio, **B** Fasting plasma glucose, and **C** Hemoglobin A1c

In individuals with diabetes, 94 (4.5%) patients died during hospitalization. From quartile 1 to quartile 4 of fasting SHR, in-hospital mortality rate increased gradually from 2.0 to 9.7% **(**Fig. 2A**)**. Patients in quartile 4 had a significantly higher rate of in-hospital mortality compared with those in quartile 1 (OR 5.145, 95%CI 2.650–9.987). In multivariable logistic regression analysis, this significant association was not changed between quartile 4 and quartile 1 (adjusted OR 4.070, 95% CI 2.014–8.228). There was no significant difference between quartile 2, quartile 3 and quartile 1 in terms of in-hospital mortality. In addition, fasting SHR was also correlated with higher in-hospital mortality when treated as a continuous variable (adjusted OR 3.682, 95% CI 2.380–5.696) **(**Table 2**)**.

**Table 2:** Association between fasting SHR and in-hospital mortality in patients with and without diabetes

Category	Event, n/total (%)	Odds ratio (95% confidence interval)	
		Unadjusted model	Multivariable-adjusted model
Diabetes			
Q1	11/537 (2.0)	Reference	Reference
Q2	13/518 (2.5)	1.231 (0.546, 2.773)	1.263 (0.543, 2.938)
Q3	19/501 (3.8)	1.885 (0.888, 4.002)	1.867 (0.849, 4.108)
Q4	51/525 (9.7)	5.145 (2.650, 9.987)	4.070 (2.014, 8.228)
SHR as a continuous variable	94/2081 (4.5)	4.815 (3.279, 7.072)	3.682 (2.380, 5.696)
No diabetes			
Q1	18/817 (2.2)	Reference	Reference
Q2	12/798 (1.5)	0.678 (0.324, 1.416)	0.706 (0.327, 1.523)
Q3	31/814 (3.8)	1.757 (0.975, 3.168)	1.532 (0.823, 2.854)
Q4	70/798 (8.8)	4.268 (2.518, 7.234)	2.976 (1.695, 5.224)
SHR as a continuous variable	131/3227 (4.1)	1.111 (0.997, 1.237)	1.109 (1.016, 1.211)

Covariates in multivariable-adjusted models were age, gender, body mass index, ST-segment elevation myocardial infarction, Killip class II/III/IV, primary percutaneous coronary intervention, current smoking, hypertension, previous myocardial infarction, previous percutaneous coronary intervention, previous stroke, chronic kidney disease, heart rate, systolic blood pressure, left ventricular ejection fraction, triglyceride, low-density lipoprotein cholesterol, and use of statin during hospitalization. *SHR* stress hyperglycemia ratio

In individuals without diabetes, 131 (4.1%) patients died during hospitalization with in-hospital mortality rates of 2.2%, 1.5%, 3.8%, and 8.8% from quartile 1 to quartile 4 of fasting SHR, respectively **(**Fig. 2A**)**. Fasting SHR as a continuous variable (adjusted OR 1.109, 95% CI 1.016–1.211) or categorical variable (Quartile 4 vs. Quartile 1: adjusted OR 2.976, 95% CI 1.695–5.224) was an independent predictor of in-hospital mortality after fully adjusting for multiple confounders. However, there was no significant difference between quartile 2, quartile 3 and quartile 1 regarding of in-hospital mortality **(**Table 2**)**.

In terms of FPG, individuals in quartile 4 had a significantly higher rate of in-hospital mortality compared with those in quartile 1 in patients with diabetes (9.4% vs. 1.9%; adjusted OR 5.354, 95% CI 2.541–11.282) and without diabetes (8.3% vs. 2.1%; adjusted OR 2.948, 95% CI 1.654–5.254). In addition, individuals in quartile 3 had a significantly higher rate of in-hospital mortality compared with those in quartile 1 in nondiabetic patients (4.6% vs. 2.1%; adjusted OR 2.032, 95% CI 1.097–3.762). Moreover, FPG as a continuous variable was positively associated with in-hospital mortality even after fully adjusting for potential confounders regardless of glucose metabolism status **(**Fig. 2B and Table 3**)**. In contrast, no statistically significant correlation between HbA1c levels, either as a continuous variable or a categorical variable, and in-hospital mortality was found in diabetic or nondiabetic patients **(**Fig. 2C and Table 4**)**.

**Table 3:** Association between FPG and in-hospital mortality in patients with and without diabetes

Category	Event, n/total (%)	Odds ratio (95% confidence interval)	
		Unadjusted model	Multivariable-adjusted model
Diabetes			
Q1	10/518 (1.9)	Reference	Reference
Q2	18/522 (3.4)	1.814 (0.829, 3.969)	2.005 (0.883, 4.554)
Q3	17/520 (3.3)	1.717 (0.779, 3.786)	1.878 (0.813, 4.338)
Q4	49/521 (9.4)	5.274 (2.641,10.531)	5.354 (2.541,11.282)
FPG as a continuous variable	94/2081 (4.5)	1.136 (1.099, 1.174)	1.125 (1.083, 1.169)
No diabetes			
Q1	17/806 (2.1)	Reference	Reference
Q2	10/806 (1.2)	0.583 (0.265, 1.281)	0.646 (0.286, 1.459)
Q3	37/808 (4.6)	2.227 (1.244, 3.989)	2.032 (1.097, 3.762)
Q4	67/807 (8.3)	4.202 (2.445, 7.223)	2.948 (1.654, 5.254)
FPG as a continuous variable	131/3227 (4.1)	1.227 (1.162, 1.295)	1.161 (1.093, 1.233)

Covariates in multivariable-adjusted models were age, gender, body mass index, ST-segment elevation myocardial infarction, Killip class II/III/IV, primary percutaneous coronary intervention, current smoking, hypertension, previous myocardial infarction, previous percutaneous coronary intervention, previous stroke, chronic kidney disease, heart rate, systolic blood pressure, left ventricular ejection fraction, triglyceride, low-density lipoprotein cholesterol, and use of statin during hospitalization. *FPG* fasting plasma glucose

**Table 4:** Association between HbA1c and in-hospital mortality in patients with and without diabetes

Category	Event, n/total (%)	Odds ratio (95% confidence interval)	
		Unadjusted model	Multivariable-adjusted model
Diabetes			
Q1	26/536 (4.9)	Reference	Reference
Q2	19/494 (3.8)	0.785 (0.429, 1.436)	0.899 (0.470, 1.720)
Q3	20/530 (3.8)	0.769 (0.424, 1.396)	0.827 (0.435, 1.573)
Q4	29/521 (5.6)	1.156 (0.671, 1.992)	1.374 (0.742, 2.544)
HbA1c as a continuous variable	94/2081 (4.5)	1.058 (0.946, 1.184)	1.099 (0.971, 1.243)
No diabetes			
Q1	38/757 (5.0)	Reference	Reference
Q2	30/769 (3.9)	0.768 (0.471, 1.253)	0.901 (0.527, 1.541)
Q3	25/815 (3.1)	0.599 (0.358, 1.002)	0.596 (0.342, 1.040)
Q4	38/886 (4.3)	0.848 (0.535, 1.344)	0.787 (0.470, 1.317)
HbA1c as a continuous variable	131/3227 (4.1)	0.782 (0.565, 1.081)	0.757 (0.538, 1.065)

Covariates in multivariable-adjusted models were age, gender, body mass index, ST-segment elevation myocardial infarction, Killip class II/III/IV, primary percutaneous coronary intervention, current smoking, hypertension, previous myocardial infarction, previous percutaneous coronary intervention, previous stroke, chronic kidney disease, heart rate, systolic blood pressure, left ventricular ejection fraction, triglyceride, low-density lipoprotein cholesterol, and use of statin during hospitalization. *HbA1c* hemoglobin A1c

### Predictive value of fasting SHR, FPG and HbA1c for in-hospital mortality

**Fig. 3:**
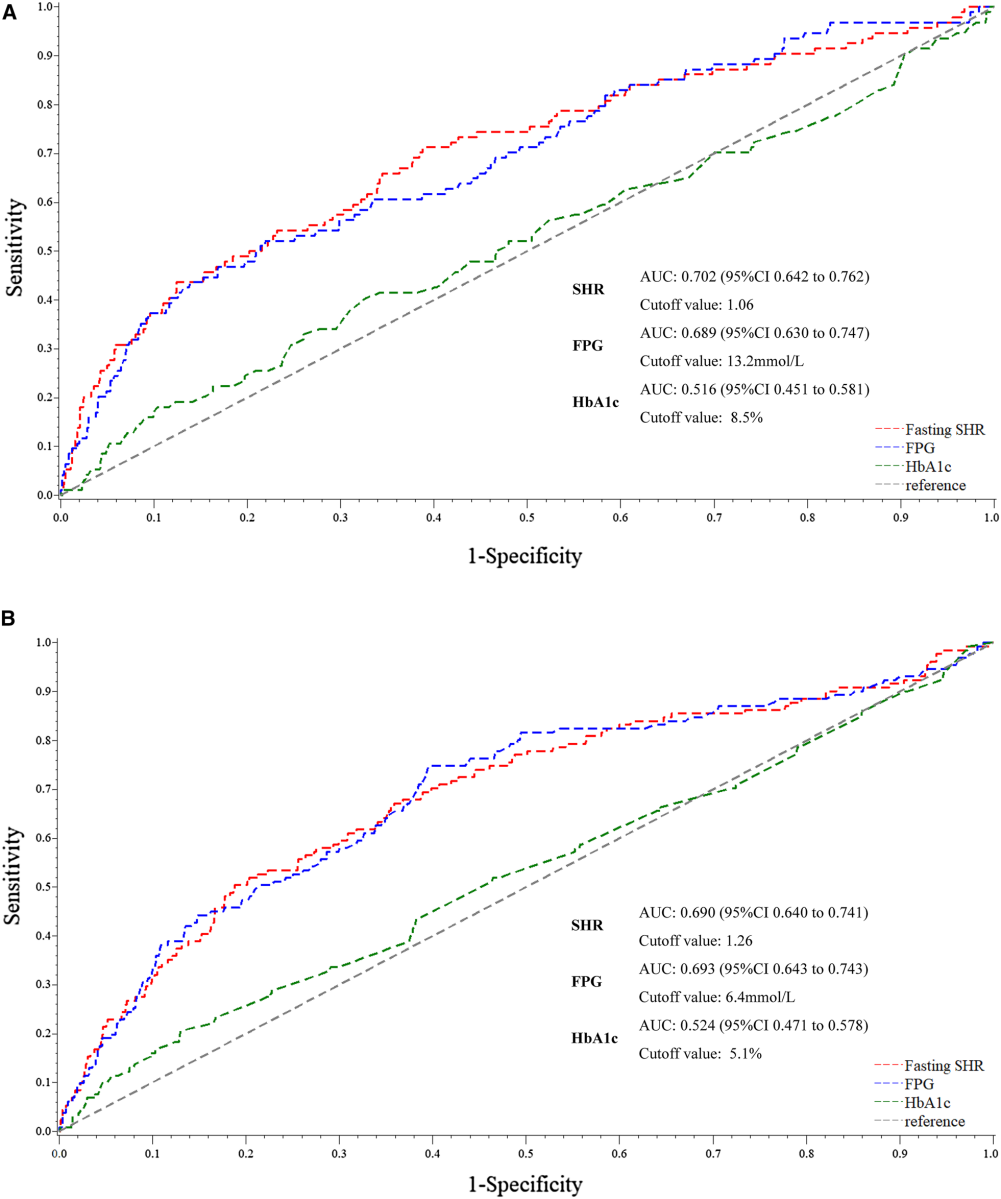
Comparison of the prognostic power of fasting SHR, FPG and HbA1c in patients **A** with diabetes and **B** without diabetes. *AUC* area under curve, *CI* confidence interval, *FPG* fasting plasma glucose, *HbA1c* hemoglobin A1c, *SHR* stress hyperglycemia ratio

At the ROC curve analysis, fasting SHR and FPG had a moderate predictive value for in-hospital mortality in patients with diabetes (AUC for fasting SHR: 0.702; FPG: 0.689) and without diabetes (AUC for fasting SHR: 0.690; FPG: 0.693) **(**Fig. 3**)**. Furthermore, the AUC for fasting SHR was not significantly different from that of FPG in diabetic patients (P = 0.471) and nondiabetic patients (P = 0.835). However, based on ROC curve analysis, the predictive value of HbA1c for in-hospital mortality was not statistically significant regardless of glucose metabolism status (P > 0.05).

### Comparative incremental predictive value of different measures of hyperglycemia

C-statistic values for the logistic prediction model of traditional risk factors were 0.821 (95% CI 0.778–0.863) and 0.856 (95% CI 0.826–0.885) for diabetic patients and nondiabetic patients, respectively. In patients with and without diabetes, adding fasting SHR (ΔC-statistic for diabetes: 0.042, 95%CI 0.016 to 0.067; No diabetes: 0.002, 95%CI 0.001, 0.004) or FPG (ΔC-statistic for diabetes: 0.038, 95%CI 0.013 to 0.063; No diabetes: 0.015, 95%CI 0.006, 0.023) to the original model resulted in a significant improvement in C-statistic **(**Table 5**)**.

**Table 5:** C-statistics for discrimination ability of different measures of hyperglycemia for in-hospital mortality

	C-Statistic (95% CI)	ΔC-statistic (95% CI)	P value
Diabetic patients			
Established risk factors	0.821 (0.778, 0.863)	Reference	
Established risk factors + SHR	0.862 (0.827, 0.897)	0.042 (0.016, 0.067)	0.002
Established risk factors + FPG	0.858 (0.823, 0.894)	0.038 (0.013, 0.063)	0.003
Established risk factors + HbA1c	0.823 (0.780, 0.865)	0.002 (-0.004, 0.009)	0.500
Nondiabetic patients			
Established risk factors	0.856 (0.826, 0.885)	Reference	
Established risk factors + SHR	0.858 (0.829, 0.888)	0.002 (0.001, 0.004)	0.001
Established risk factors + FPG	0.871 (0.841, 0.899)	0.015 (0.006, 0.023)	0.0008
Established risk factors + HbA1c	0.855 (0.826, 0.885)	-0.001 (-0.004, 0.003)	0.715

Original model included age, sex, body mass index, ST-elevation myocardial infarction, Killip class II/III/IV, primary percutaneous coronary intervention, current smoking, hypertension, previous myocardial infarction, previous percutaneous coronary intervention, previous stroke, chronic kidney disease, heart rate, systolic blood pressure, left ventricular ejection fraction, triglyceride, low-density lipoprotein cholesterol, and use of statin during hospitalization. *CI* confidence interval, *FPG* fasting plasma glucose, *HbA1c* hemoglobin A1c, *SHR* stress hyperglycemia ratio

## Discussion

### Main findings

This study, for the first time, compared the prognostic effect of fasting SHR, FPG and HbA1c for in-hospital mortality in patients with AMI. High fasting SHR and FPG, rather than HbA1c, were significantly associated with higher in-hospital mortality in AMI patients with or without diabetes. In addition, the prognostic power of fasting SHR did not differ from FPG in both diabetic and nondiabetic patients. Harrell’s C-statistic further proved that fasting SHR and FPG could improve the risk prediction for in-hospital mortality in a model of traditional risk factors in this population.

### Mechanism and detrimental effects of stress hyperglycemia

In the setting of AMI, the increasing levels of glucagon, cortisol, and cytokine promote the production of glucose by upregulation of gluconeogenesis and glycogenolysis [26–28]. However, the impaired insulin secretion by pancreatic β-cell could not overcome the hyperglycemic effects of these counter-regulatory hormones and cytokines, leading to the incidence of stress hyperglycemia [29, 30]. What’s worse, the activation of sympathetic nervous system evokes insulin resistance through mobilizing free fatty acids (FFAs) from adipose tissue and stimulating serine/threonine kinases that interfere with the insulin signaling [31, 32]. Generally, stress hyperglycemia triggers inflammation and oxidative stress, aggravates endothelial dysfunction, induces a prothrombotic state, and leads to impaired coronary flow, increased infarct size, and poor cardiac function. For example, a study with 460 patients with STEMI showed that patients with hyperglycemia were less often to have Thrombolysis In Myocardial Infarction (TIMI) flow grade 3 before primary PCI (12% vs. 28%, P < 0.001) [33]. Iwakura and colleagues showed a higher incidence of no-reflow phenomenon (52% vs. 14%, P < 0.001), higher peak creatine kinase level (2.50 vs. 1.80 IU/L, P = 0.005) and lower change in the wall motion score (3.7 vs. 5.7, P = 0.01) in patients with hyperglycemia after primary PCI [34]. Moreover, Kersten and colleagues reported that the impairment of collateral circulation induced by hyperglycemia was associated with increased infarct size [35, 36]. Furthermore, the reduced glycolytic substrate and excessive FFAs caused by insulin deficiency may reduce myocardial contractility and increase the risk of pump failure and arrhythmia [37].

### Findings and shortcomings of previous studies

However, no uniform definition for stress hyperglycemia has been established at present. In 2015, Roberts and colleagues proposed SHR to better identify stress hyperglycemia through quantifying the magnitude of a relative glycemic rise from chronic glycemia of the past 2 ~ 3 months in patients at risk of critical illness [11]. A study with 2875 non-surgical hospitalized patients with heart failure and diabetes showed that people with SHR in tertile 3 presented higher risks of composite cardiovascular events (death, cardiopulmonary resuscitation, cardiogenic shock, or acute heart failure) than those with SHR in tertile 2 (OR 1.89, 95% CI 1.26–2.87), while people with SHR in tertile 1 had a statistically non-significantly increased risk of cardiovascular events than those with SHR in tertile 2 (OR 1.23, 95% CI 0.79–1.93) [2]. Xu and colleagues reported that SHR may be an effective predictor of in-hospital mortality in patients with coronary artery disease, especially for those with pre-diabetes and diabetes [38]. Actually, there were many studies to explore the effect of SHR on prognosis in patients with AMI. Nevertheless, the association between SHR and short- or long-term mortality has not been well established in this population. Some studies reported that SHR was significantly associated with poor prognosis [12–14], whereas others did not [15, 16]. One of the possible reason for the controversial results is that conventional SHR, as calculated from admission glucose and HbA1c, may also be affected by the timing of meal.

### Strengths of the present study

In the present study, patients in the highest quartile of fasting SHR had a higher rate of in-hospital mortality than that in the lowest quartile, both in diabetic and nondiabetic cohorts. Moreover, fasting SHR had a moderate discrimination ability for in-hospital mortality in patients with AMI (AUC for diabetes: 0.702; No diabetes: 0.690), which appeared to be stronger than conventional SHR. In a study with 1300 STEMI patients treated with PCI, conventional SHR presented a weak albeit statistically significant discrimination ability for in-hospital death, cardiogenic shock or acute pulmonary edema (AUC for diabetes: 0.63, 95% CI 0.56–0.70; No diabetes: 0.67, 95% CI 0.58–0.75) [39]. Similarly, Schmitz and colleagues found that in diabetic patients with AMI, the AUC for conventional SHR was 0.64 (96% CI 0.56–0.73) for 28-day mortality and 0.59 (96% CI 0.53–0.65) for 5-year mortality [40]. Actually, our previous study has demonstrated that high fasting SHR were significantly associated with higher in-hospital mortality compared with those with low fasting SHR in diabetic and nondiabetic patients with AMI. However, patients were only divided into two groups, and the relationship between fasting SHR and prognosis could not be fully explored [18]. In a study with 2089 AMI patients, Luo and colleagues also reported that individuals with high fasting SHR were significantly associated with poor prognosis in AMI patients with different metabolism status. Moreover, adding fasting SHR to the GRACE score significantly improved its diagnostic performance (integrated discrimination improvement and net reclassification improvement) in patients with diabetes [41]. However, the GRACE score is generally used in patients with NSTEMI or unstable angina, and is not applicable to patients with STEMI. Moreover, the above two studies did not compare fasting SHR with FPG and HbA1c in AMI patients [18, 41].

In addition to fasting SHR, FPG can also rule out the influence of diet on stress hyperglycemia. However, limited information was available about the clinical significance of FPG in patients with AMI. Suleiman and colleagues found that, compared with normal FPG, the adjusted OR for 30-day mortality progressively increased with higher tertiles of elevated FPG in individuals with nondiabetic AMI patients. In addition, patients with normal admission glucose and elevated FPG, rather than those with elevated admission glucose and normal FPG, had a statistically significant higher rate of 30-day mortality compared with those with normal FPG and admission glucose, indicating that FPG was superior to admission glucose in the assessment of short-term risk [17]. However, the prognostic effect of fasting SHR for in-hospital mortality in AMI patients with diabetes had not been evaluated in that study. Notably, this study firstly indicated that FPG had a similar predictive value for in-hospital mortality compared with fasting SHR in diabetic and nondiabetic patients.

### Implications for treatment of stress hyperglycemia

Although some studies reported that insulin-based tight glycemic control may provide potential benefits to the ischemic myocardium [42], most randomized trials did not find a lower rate of short- and long-term mortality in AMI patients with hyperglycemia who received insulin therapy. Marfella and colleagues reported that individuals with intensive glycemic control had lower oxidative stress and inflammation than those with conventional glycemic control [43]. The DIGAMI (Diabetes Mellitus, Insulin Glucose Infusion in Acute Myocardial Infarction) 1 trial proved that insulin-glucose infusion followed by intensive subcutaneous insulin in 620 diabetic AMI patients improved 1- and 3.4-year survival [44]. On the contrary, the DIGAMI 2 trial did not support that an acutely introduced, long-term insulin treatment reduced in-hospital or 2.1-year mortality in 1253 diabetic patients with AMI [45]. In addition, the HI-5 trial showed that intensive insulin therapy was not associated with lower rate of in-hospital or long-term mortality in hyperglycemic patients without previously established diabetes [46]. However, admission glucose was used to define stress hyperglycemia in those studies, which could not fully reflect the acute glycemic rise. This study suggested that fasting SHR or FPG may be better to identify true hyperglycemic patients who will benefit from intensive treatment strategies. In recent years, glucagon-like peptide-1 receptor agonists (GLP-1 RAs) and sodium-glucose co-transporters 2 inhibitors (SGLT-2Is) have been proved to reduce glucose level and risk of cardiovascular events in patients with hyperglycemia [47]. Therefore, a combined therapy of long-acting GLP-1 RAs or SGLT-2Is with basal insulin, rather than insulin therapy alone, may be the right choice for these patients.

### Limitations

There are some limitations in this study. First, this is a secondary analysis of the prospective, nationwide, multicenter CAMI registry. Although multivariable-adjusted analysis were conducted, it was impossible to control all the confounding factors. Thus, the findings should be interpreted as hypothesis generating. Second, it was difficult for us to rule out selection bias, as only participants with PFG and HbA1c levels were included. Third, patients were stratified based on the presence of diabetes in this study. Prediabetes is an intermediate state between diabetes and normal glucose metabolism. However, due to the limitation of sample size, we did not investigate the relationship between fasting SHR and in-hospital mortality in prediabetic patients exclusively. Fourth, this study did not compare the prognostic effect of fasting SHR and conventional SHR for in-hospital mortality, as admission glucose was not collected in Phase II of the CAMI registry. Fifth, both fasting SHR and FPG are derived from one blood glucose test, which cannot reflect the full profile of glucose swings in the setting of AMI. Therefore, the association between glucose fluctuation identified by continuous glucose monitoring system and prognosis need to be assessed in the future. Last but not least, the conclusions of this study cannot be directly extrapolated to non-Asian populations without validation.

## Conclusions

This study indicated that, in individuals with AMI, fasting SHR as well as FPG was strongly associated with in-hospital mortality regardless of glucose metabolism status. Fasting SHR and FPG might be considered as a useful marker for risk stratification in this population.

## Supplementary Information

**Additional file 1: Table S1.** Members of committees and teams. **Table S2.** Investigators in the CAMI registry. **Table S3.** Baseline characteristics of patients with diabetes according fasting SHR  levels. **Table S4.** Baseline characteristics of patients without diabetes according fasting SHR levels.

## Data Availability

The datasets used and/or analyzed during the current study are available from the corresponding author on reasonable request.

## Abbreviations

AMI: Acute myocardial infarction
AUC: Areas under the curve
CAMI: China acute myocardial infarction
CI: Confidence interval
DIGAMI: Diabetes mellitus, insulin glucose infusion in acute myocardial infarction
FFA: Free fatty acid
FPG: Fasting plasma glucose
eCRF: Electronic case report form
GLP-1 RA: Glucagon-like peptide-1 receptor agonist
GRACE: Global registry of acute coronary event
HbA1c: Glycosylated hemoglobin A1c
LVEF: Left ventricular ejection fraction
NSTEMI: Non-ST-segment elevation myocardial infarction
OR: Odds ratio
PCI: Percutaneous coronary intervention
ROC: Receiver operating characteristic
SGLT-2I: Sodium-glucose co-transporters 2 inhibitor
SHR: Stress hyperglycemia ratio
STEMI: ST-segment elevation myocardial infarction
TIMI: Thrombolysis in myocardial infarction
